# Intravascular Ultrasound vs. Fractional Flow Reserve for Percutaneous Coronary Intervention Optimization in Long Coronary Artery Lesions

**DOI:** 10.3390/diagnostics13182921

**Published:** 2023-09-12

**Authors:** Povilas Budrys, Aaron Peace, Arvydas Baranauskas, Giedrius Davidavicius

**Affiliations:** 1 Clinic of Cardiac and Vascular Diseases, Faculty of Medicine, Vilnius University, 03101 Vilnius, Lithuania; 2 Cardiology and Angiology Center, Vilnius University Hospital Santaros Klinikos, 08661 Vilnius, Lithuania; 3 Department of Cardiology, Western Health and Social Care Trust, Derry BT47 6SB, UK

**Keywords:** fractional flow reserve, FFR, intravascular ultrasound, IVUS, long lesions, diffuse disease

## Abstract

Background: intravascular ultrasound (IVUS) and fractional flow reserve (FFR) have both been shown to be superior to angiography in optimizing percutaneous coronary intervention (PCI). However, there is still a lack of comparative studies between PCI optimization using physiology and intravascular imaging head-to-head. The aim of this study was to compare the effectiveness of FFR and IVUS PCI optimization strategies on the functional PCI result (assessed with FFR) immediately post-PCI and at 9–12 months after the treatment of long coronary lesions. Methods: This was a single-center study comparing post-PCI FFR between two different PCI optimization strategies (FFR and IVUS). The study included 154 patients who had hemodynamically significant long lesions, necessitating a stent length of 30 mm or more. The procedural outcomes were functional PCI result immediately post-PCI and at 9–12 months after treatment. Clinical outcomes included target vessel failure (TVF) and functional target vessel restenosis rate during follow-up. Results: Baseline clinical characteristics and FFR (0.65 [0.55–0.71]) did not differ significantly between the two groups and the left anterior descending artery was treated in 82% of cases. The FFR optimization strategy resulted in a significantly shorter stented segment (49 mm vs. 63 mm, *p* = 0.001) compared to the IVUS optimization strategy. Although the rates of optimal functional PCI result (FFR > 0.9) did not significantly differ between the FFR and IVUS optimization strategies, a proportion of patients in the FFR group (12%) experienced poor post-PCI functional outcome with FFR values ≤ 0.8, which was not observed in the IVUS group. At the 9–12 month follow-up, 20% of patients in the FFR group had target-vessel-related myocardial ischemia, compared to 6% in the IVUS group. The rates of TVF and functional target vessel restenosis during follow-up were also numerically higher in the FFR optimization group. Conclusions: The use of FFR PCI optimization strategy in the treatment of long coronary artery lesions is associated with a higher incidence of poor functional PCI result and larger myocardial ischemia burden at follow-up compared to the IVUS optimization strategy. However, this discrepancy did not translate into a statistically significant difference in clinical outcomes. This study highlights the importance of using IVUS to optimize long lesions functional PCI outcomes.

## 1. Background

Percutaneous coronary intervention (PCI) of long coronary artery lesions is often challenging and associated with a suboptimal functional result and an increased likelihood of target vessel failure (TVF) [1,2,3]. As a result, bypass surgery is often chosen as the preferred revascularization strategy for long diffuse segments of atheromatous disease, yet there is a lack of evidence to support this strategy.

Although FFR is considered to be the ‘gold standard’ to determine the functional significance of a lesion before PCI, it is not routinely measured post-PCI to evaluate the functional result. This is in the face of evidence demonstrating a strong link between FFR values ≤ 0.80 post-PCI and adverse outcomes [4,5,6,7]. Furthermore, angiographically successful PCI measured by the human eye is frequently not desirable when residual ischemia is still present post-PCI in as many as 30% of cases [1,8,9,10]. Given that these disappointing results were found in short- to medium-length lesions, it is likely that the numbers of cases with residual ischemia post-PCI will be proportionately higher in long diffuse coronary lesions [1].

Intravascular ultrasound (IVUS) and fractional flow reserve (FFR) optimized PCI have both been proven to be superior to angiography-only guided PCI [8,11,12,13,14,15,16]. TARGET FFR trial showed that if FFR is used post-PCI for optimization that the proportion of patients with a post-PCI FFR of ≤ 0.80 is reduced by 11.2% [8]. Similarly, the DOCTORS study, which randomized optical coherence tomography (OCT)-guided PCI to angiography-guided PCI, found that post-PCI FFR was significantly higher in the OCT group (0.94 ± 0.04 versus 0.92± 0.05, *p* = 0.005) [17]. However, there is still a lack of studies comparing physiology to intravascular imaging head-to-head particularly in the setting of long diffuse coronary artery disease.

Therefore, we sought to compare the FFR and IVUS PCI optimization strategies on the functional PCI result immediately post-PCI and at 9–12 months after the treatment of long coronary artery lesions.

## 2. Methods

This was a single-center trial comparing post-PCI FFR between two different PCI optimization strategies (FFR and IVUS) in the PCI of long coronary lesions. A total of 154 patients with stable coronary artery disease or acute coronary syndrome without ST segment elevation (NSTE-ACS) with hemodynamically significant (FFR ≤ 0.80) long lesions necessitating a stent length of ≥30 mm were included.

The study employed a two-stage approach involving two separate cohorts to assess the impact of different PCI optimization strategies on post-PCI FFR (quasi-experimental sequential cohort study).

### 2.1. Stage 1: FFR-Optimized Cohort

A cohort of 74 consecutive patients was enrolled for the FFR-optimized group. These patients underwent FFR-optimized PCI (see FFR-optimized protocol below). Patients who met the following criteria were included in the study: age over 18, diagnosed with chronic coronary syndrome or NSTE-ACS (including unstable angina or myocardial infarction without ST segment elevation), and had hemodynamically significant lesions (with FFR of 0.8 or lower) necessitating a stent length of 30 mm or more and suitable for PCI. Patients were excluded if they had ST segment elevation acute myocardial infarction, presence of chronic total occlusion, contraindications for dual antiplatelet therapy, a life expectancy of one year or less, or were allergic to everolimus, biolimus, sirolimus, or zotarolimus.

### 2.2. Stage 2: IVUS-Optimized Cohort

Following the completion of stage 1, a second stage was conducted with 80 consecutive patients (referred to as the IVUS-optimized group). These patients met the same inclusion and exclusion criteria as the FFR-optimized group. However, in this stage, patients underwent IVUS-optimized PCI (see IVUS-optimized protocol below).

**Figure 1:**
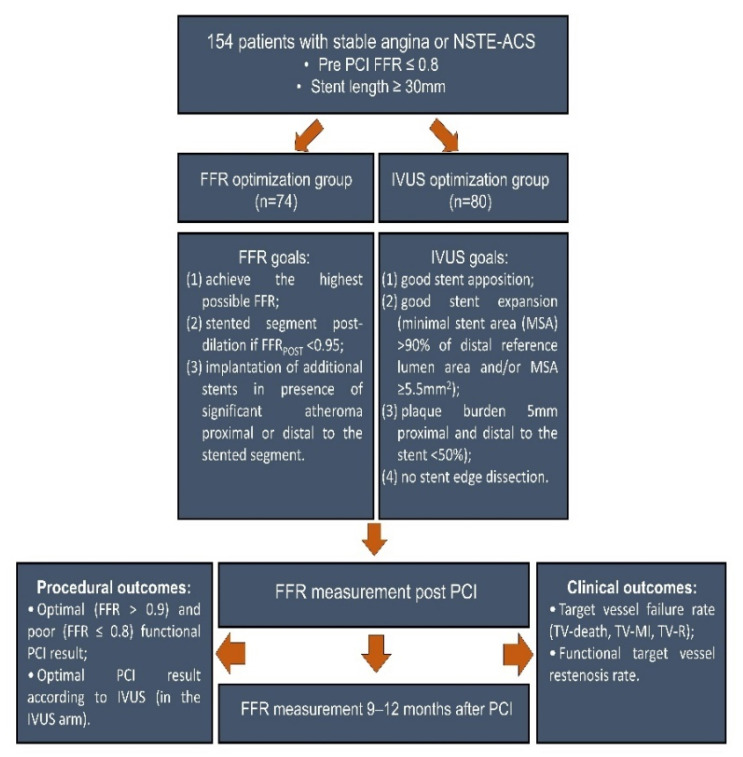
Clinical trial flow chart.

Patients from both groups underwent invasive follow-up with repeat FFR measurement after 9–12 months since initial PCI (Figure 1).

The research protocol for this study has been approved by the ethics committee in accordance with the principles outlined in the Declaration of Helsinki (approval number Nr.2019/6-1150-639). All participating patients provided informed consent by signing the necessary forms. The trial has been registered on ClinicalTrials.gov (identifier: NCT05732324).

## 3. Fractional Flow Reserve Protocol

Fractional flow reserve protocol was applied for all patients. FFR measurement was conducted following established protocol. To induce maximal hyperemia, adenosine was administered intravenously at a rate of 140 μg/kg/min, preceded by the administration of 200 mcg of intracoronary nitrates. FFR values were obtained using a coronary pressure wire (Abbott Vascular, Plymouth, MN, USA), and a value of 0.8 or lower was deemed indicative of functional significance. FFR measurements were taken both before and after PCI, specifically at the distal third of the coronary artery.

Pullbacks were performed before and after PCI. In the FFR-optimized group, more than one post-PCI FFR measurement could be acquired if the operators performed additional optimization. In the IVUS-optimized group, only one post-PCI FFR measurement was recorded after which the procedure was considered to be completed, and no further interventions were undertaken. The same FFR measurements were performed at 9–12 months follow-up.

## 4. FFR-Optimized Protocol

The goal was to achieve the highest possible FFR. The operators were encouraged to perform post-dilatation in all cases. If FFR post-PCI was < 0.95, further post-dilatation was mandatory. During the pull-back analysis, if a significant focal change in the gradient was observed, additional interventions were carried out accordingly. If the gradient change was observed within the stented segment, additional post-dilatation was performed. Conversely, if the gradient change occurred in the non-stented segment, additional stent was implanted. However, if a gradual change in the gradient was observed, indicative of diffuse disease distal to the stented segment, in-stent, or proximal to the stented segment, no further interventions were undertaken. The final FFR measurement was obtained at the conclusion of the procedure, once both the angiographic and functional outcomes were deemed satisfactory. Importantly, no further interventions were carried out following the final FFR measurement.

## 5. IVUS-Optimized Protocol

**Figure 2:**
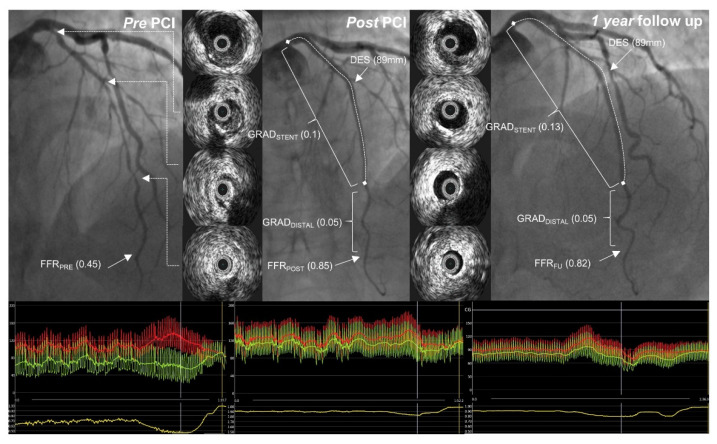
A case example. Pre-, post-percutaneous coronary intervention (PCI) and one-year follow-up angiographic images with intravascular ultrasound pictures from a corresponding left anterior descending artery segment. Fractional flow reserve curves are presented below. FFR_PRE_—FFR before PCI; FFR_POST_—FFR post-PCI; GRAD_DISTAL_—distal gradient (from the distal segment to the distal stent edge); GRAD_STENT_—trans-stent gradient; FFR_FU_—FFR recorded at follow-up.

Before performing PCI, IVUS was utilized to determine the ideal stent implantation spots, aiming for locations with a plaque volume of less than 50%. The stent diameter was selected based on the distal external elastic membrane diameter minus 0.25 mm (Figure 2). IVUS also aided operators in selecting the proper tools for lesion pre-dilatation. The Eagle Eye Platinum IVUS catheter (Philips, Cambridge, MA, USA) was employed for IVUS procedures.

**Figure 3:**
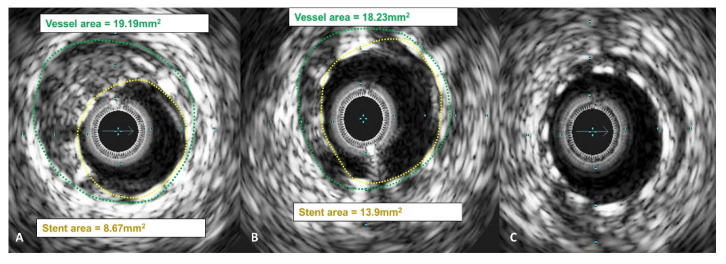
IVUS images showing stent expansion and apposition. (**A**) Good absolute stent expansion according to the stent area, however, suboptimal relative stent expansion according to the vessel area; (**B**) improved absolute and relative stent expansion after post-dilating stent with a larger balloon; (**C**) good stent expansion. All images demonstrate good stent apposition.

**Figure 4:**
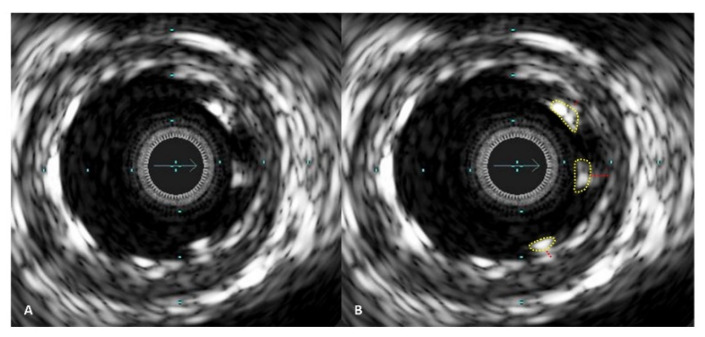
IVUS images demonstrating stent malapposition. In picture (**A**), malapposed stent struts are visible on one side of the artery. In picture (**B**), the malapposed stent struts are marked with a yellow line, and the red lines indicate the distance between the stent struts and the vessel wall inner layer.

Operators aimed to achieve an optimal anatomical outcome during PCI, guided by IVUS assessment using the following criteria: (1) ensuring proper stent apposition, (2) achieving adequate stent expansion (with a minimal stent area (MSA) greater than 90% of the distal reference lumen area and/or MSA of at least 5.5 mm^2^), (3) maintaining a plaque burden below 50% within 5 mm proximally and distally to the stent, and (4) avoiding stent edge dissection. A case example demonstrating stent expansion and apposition is demonstrated in Figure 3 and Figure 4.

Following stent optimization, a final IVUS run was conducted. The IVUS run was regarded conclusive when no additional optimization was deemed feasible. At that stage, post-PCI FFR measurement was performed, and regardless of the obtained FFR value, no additional interventions were undertaken. An optimal anatomical outcome was defined as meeting all four IVUS criteria.

## 6. Study Outcomes

Procedural and clinical outcomes were compared between FFR-optimized and IVUS-optimized PCI groups (Figure 1).

Procedural outcomes of the study: optimal functional PCI result (FFR > 0.9) and poor functional PCI result (FFR ≤ 0.8) immediately post-PCI and at 9–12 months follow-up; optimal PCI result according to IVUS (only in the IVUS-optimized group).

Clinical outcomes of the study: target vessel failure rate (target-vessel-related death (TV-death), target-vessel-related spontaneous myocardial infarction (TV-MI), any target vessel revascularization (TV-R)) during one-year follow-up; functional target vessel restenosis rate at 9–12 months follow-up.

TV-death—all instances of cardiac death, unless substantial evidence was present to indicate alternative causes.

TV-MI was determined based on clinical symptoms, ECG changes, and/or imaging findings indicative of MI. Additionally, an elevation of TnI or TnT levels beyond the 99th percentile of the upper normal limit, along with the identification of a culprit lesion within the target vessel through coronary angiography, was required.

TV-R encompassed any revascularization performed on the previously treated vessel.

Functional TV restenosis was defined as an FFR value of 0.80 or lower observed during the 9–12 months follow-up. Patients with an FFR value of 0.80 or lower immediately after PCI were excluded from the follow-up analysis regarding functional restenosis.

## 7. Statistical Analysis

Mean values (±standard deviation) or median values [Q1–Q3] were used to express continuous variables, depending on their distribution. Continuous variables with a normal distribution were compared using a Student’s *t*-test, while the nonparametric Mann–Whitney U test was utilized for variables that did not follow a normal distribution. Categorical variables were presented as frequencies and compared using the chi-square test.

## 8. Results

Baseline clinical characteristics did not differ significantly between the FFR-optimized and IVUS-optimized groups (Table 1), apart from there being fewer patients with previous non-target-vessel-related myocardial infarction (MI) in the FFR-optimized group (39.2% vs. 57.5% *p* = 0.02). The average age was 66 years, predominantly male (72%), with the majority of patients (75%) undergoing PCI for chronic coronary syndrome. Patients from both groups had a normal left ventricle ejection fraction, with the median being lower in the FFR-optimized group (50% vs. 55%, *p* = 0.02).

## 9. Results Associated with PCI

Left anterior descending artery (LAD) was the most commonly treated vessel (82%) (Table 2). FFR optimization strategy resulted in less frequent coverage of the left main artery during LAD treatment (3% vs. 16%, *p* = 0.01). Pre-dilatation was used routinely in both groups, however, smaller diameter balloons were used in the FFR-optimized group (2.5 [2.2–2.8] mm vs. 2.6 [2.5–2.9], *p* = 0.001). The FFR-optimized group had significantly shorter stented segments (49 [36.0–60.3] mm vs. 63 [48.0–76.0] mm, *p* = 0.001) compared to IVUS-optimized group. Post-dilatation was performed less frequently in the FFR group again using smaller diameter balloons (3.5 [3.5–3.5] mm vs. 4.0 [3.8–4.5] mm, *p* = 0.001). The contrast volume used during procedure was similar in both groups.

## 10. Fractional Flow Reserve Findings

**Figure 5:**
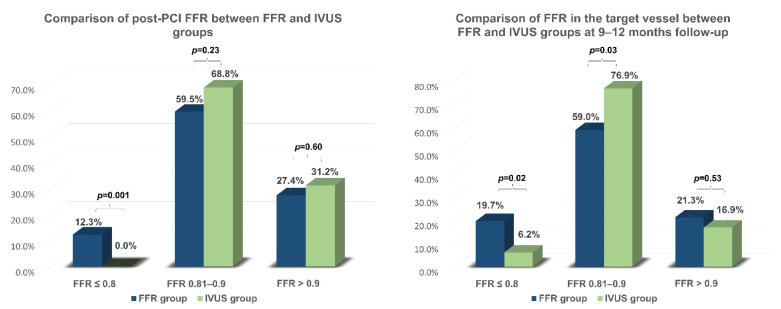
Comparison of post-PCI and 9–12 months FFR measured in the target vessel between FFR and IVUS groups. PCI—percutaneous coronary intervention; FFR—fractional flow reserve; IVUS—intravascular ultrasound.

The median pre-PCI FFR was 0.65 [0.55–0.71] and did not differ significantly between the FFR-optimized and IVUS-optimized groups (Table 3). However, post-PCI 12.3% of patients in the FFR-optimized group had FFR ≤ 0.8. In comparison, none of the patients in the IVUS-optimized group had an FFR ≤ 0.8, *p* = 0.001 (Figure 5). An optimal functional PCI result (FFR post-PCI > 0.9) was achieved in 27% of FFR-optimized group compared to 31% in the IVUS-optimized group (*p* = 0.6). The median of acute FFR gain (Δ post-PCI FFR and pre-PCI FFR) was 0.23 [0.17–0.33] and it did not differ significantly between two groups.

A total of 82% of patients underwent coronary angiography with FFR assessment at 9–12 months follow-up. The long-term FFR gain (Δ FFR at follow-up and pre-PCI FFR; Δ FFR at follow-up and post-PCI FFR) also did not differ significantly between the two groups. In the follow-up FFR pullback examination, the FFR-optimized group displayed a smaller trans-stent gradient (0.06 vs. 0.08, *p* = 0.04) but a larger distal gradient (0.04 vs. 0.03, *p* = 0.02) compared to the IVUS-optimized group. Repeat FFR measurements demonstrated larger ischemic (FFR ≤ 0.8) burden in the FFR-optimized group compared to the IVUS-optimized group (20% vs. 6%, *p* = 0.02).

## 11. Intravascular Ultrasound Findings

The results from the IVUS-optimized group are demonstrated in Table 4. IVUS analysis revealed that after stents implantation and optimization, 38% of patients needed additional optimization (either post-dilatation or additional stenting). Half of treated segments had characteristics of severe calcification. Operators managed to achieve an optimal IVUS result in 68% of cases and significant plaque volume (≥50%) near stent edges was the characteristic most often related to suboptimal IVUS result.

## 12. Medical Therapy

Every patient received antiplatelet therapy, with a higher proportion in the IVUS-optimized group requiring triple therapy. Upon discharge, the majority of patients in both groups were prescribed a statin, beta-blocker, and ACE-inhibitor or ARB, with no significant difference between the groups (Table 5).

## 13. Follow-Up Results

Functional target lesion restenosis was numerically more frequent in the FFR group (13.5% vs. 6.2% in IVUS group); however, this did not achieve a statistical significance (*p* = 0.18). There were no TV-related deaths and spontaneous myocardial infarctions in either group during one-year follow-up. Although the rate of target vessel revascularization was numerically higher in the FFR group (8.1% vs. 3.8%), it did not reach statistical significance, *p* = 0.25 (Table 6).

## 14. Discussion

The main finding of our study is a significant difference between two groups in the percentage of patients exhibiting persistent myocardial ischemia: there were 12% of patients with post-PCI FFR ≤ 0.8 in the FFR group, while there were no such patients in IVUS optimization group. The trend for higher ischemia prevalence in FFR optimization strategy continued during follow-up: there were 20% of patients with target-vessel-related myocardial ischemia in the FFR group and 6% in the IVUS group. The IVUS assessment confirmed the attainment of an optimal anatomical PCI outcome in two-thirds of the IVUS group patients. Target vessel failure and functional target vessel restenosis rate during follow-up were numerically higher in the FFR optimization group, however, this difference did not reach statistical significance.

To the best of our knowledge, this study represents the first comparison of two contemporary PCI optimization strategies (FFR vs. IVUS) in treating long coronary artery lesions. It is worth making a note of the results of a recent FLAVOUR (Fractional Flow Reserve and Intravascular Ultrasound-Guided Intervention Strategy for Clinical Outcomes in Patients With Intermediate Stenosis) trial that examined FFR-guided PCI versus IVUS-guided PCI for patients with intermediate coronary stenosis [18]. In the FFR group, PCI was performed if FFR ≤ 0.8 and considered optimized if post-PCI FFR ≥ 0.88. In the IVUS group, PCI was performed based on criteria involving minimal luminal area and plaque burden. The FLAVOUR trial concluded that FFR-guided PCI had a similar incidence of adverse cardiovascular events at 24 months compared to IVUS-guided PCI. However, there are significant differences between our study and the FLAVOUR trial. We used FFR to assess baseline ischemia and determine the need for PCI in both groups. In the IVUS-optimized group, IVUS was used solely for procedure optimization and not for determining whether to perform PCI. FFR measurements were taken post-PCI and during follow-up to objectively evaluate the PCI outcomes in both groups. Finally, our study specifically focused on long coronary artery lesions.

FFR is an important tool to identify hemodynamically significant coronary artery lesions and clear cut-offs to perform or defer coronary artery intervention are well-established in current guidelines [19]. However, post-PCI FFR assessment is not performed routinely, despite strong evidence linking post-PCI FFR to patients’ outcomes. Although there is a discrepancy regarding optimal post-PCI FFR values, many studies and meta-analyses agree that the bigger FFR, the smaller likelihood for the target vessel failure to occur. In our study, FFR was used not only before PCI to determine myocardial ischemia, but also during PCI procedure for functional optimization (in FFR-optimized group) and after PCI optimization to establish the final functional PCI result. Our results demonstrate that the median post-PCI FFR (0.88) and the rate of optimal functional PCI result (FFR > 0.9) did not differ significantly between two PCI optimization strategies; nonetheless, 12% of the FFR group patients had an ischemic post-PCI FFR (≤0.8) and there were no such patients in the IVUS group. Previous trials, which assessed post-PCI FFR in drug-eluting stents era, demonstrated that a significant number of patients (8–30%) [1,8,9,16] have residual myocardial ischemia post-PCI even after performing functional optimization [8,16]. However, a few significant differences from our study should be underlined. First, these trials consisted of significantly shorter lesions (average stent length—20–31 mm), while our focus was long coronary artery lesions (average stent length—56 mm). Secondly, the left anterior descending artery was the treated vessel in 82% of our sample cases, while this proportion was smaller (44–57%) in comparable trials. Both lesion length and LAD artery were proved to be independent predictors of poor functional PCI result [9,10]. Thus, 12% of residual ischemia cases in the FFR optimization group after treatment of long, mainly LAD lesions could be considered an acceptable result; yet the application of IVUS reduced this number even further—to zero. There are several factors associated with suboptimal functional PCI results. Stent under-expansion or malapposition, residual focal lesions, dissections near stent edges, or diffuse disease downstream can influence post-PCI FFR and it is difficult to establish these issues solely on angiogram. Post-PCI FFR recording and a pull-back could help to identify areas of significant pressure drop, which could be treated by stented segment post-dilatation or additional stent implantation, consequently improving functional PCI result as was demonstrated in the TARGET-FFR trial [8]. However, the addition of intravascular imaging could provide even more information about underlying ischemia mechanisms and possibly facilitate PCI optimization [17]. A selective approach to use intravascular imaging only for lesions with suboptimal functional outcome (FFR < 0.9 post-PCI) is a negotiable alternative in order to optimize cost-effectiveness. The effectiveness of this approach was examined in a recent study called FFR-REACT, which involved the randomization of 291 patients with post-PCI FFR values below 0.9 into two groups: IVUS-guided optimization and a control arm [20]. The study demonstrated that IVUS-guided PCI optimization resulted in a significant improvement in post-PCI FFR, with a mean increase from 0.82 ± 0.06 to 0.85 ± 0.05 (*p* < 0.001). Furthermore, the study found that 20% of the treated vessels achieved a post-PCI FFR value exceeding 0.90. However, due to lower-than-expected event rates, this strategy did not significantly reduce TVF rate at the one-year follow-up.

There were a few important differences related to PCI between the FFR and IVUS optimization strategies in our sample. The use of intravascular imaging was associated not only with more often performed lesion pre- and post-dilatation but also with bigger size of the balloons used, which corroborates with the data from previous trials [11,13]. Furthermore, the stented segment length was significantly longer in the IVUS group, and the left main artery was more frequently covered with stent during LAD treatment. These differences could be explained by the visual information received from the IVUS. The application of intravascular imaging allows precise selection of stents and balloons size, stent landing zones, and it usually demonstrates more disease than one could appreciate from the angiogram, resulting in longer stented segments.

The criteria for determining an optimal IVUS outcome resembled those utilized in the ULTIMATE trial [11], with the exception of a larger desirable MSA in our study (5.5 mm^2^ versus 5.0 mm^2^). Notably, a higher proportion of patients (68%) achieved an optimal IVUS result in our study compared to the ULTIMATE trial, where this target was met by 53% of patients. It is important to consider that while the ULTIMATE trial encompassed various lesion types, our study specifically concentrated on long diffuse coronary lesions.

Previous trials demonstrated poor results following treatment of long lesions. Honda et al. reported a one-year TVR rate of 8% after ultra-long DES implantation (>50 mm), despite routine use of IVUS [2]. Similarly, analysis from GRAND-DES registry showed a two-year TVF rate of 8.1% when >40 mm stents were used [3]. Our study revealed an identical one-year TVF rate (8.1%) in the FFR-optimized group; however, for the IVUS-optimized group, the one-year TVF rate was numerically lower—3.8%. One possible explanation for the lower TVF rate in the IVUS-optimized group could be better lesion coverage and stent expansion; however, this is a hypothesis-generating idea that requires further exploration. The smaller incidence of TVR in our IVUS-optimized group compared to Honda et al. could be partially explained by the different IVUS optimization criteria used, as they were not that strict in mentioned study, and in GRAND-DES sample, IVUS was not used routinely. Moreover, most trials perform only clinical follow-up and some TVF events might be unrecognized. In our study, the majority of patients underwent repeat coronary angiogram with FFR measurement during follow-up, consequently, our reported TVF rate could be higher compared to a real-life setting, as some TVR may occur in asymptomatic patients.

Treating long diffuse coronary artery disease by whatever revascularization strategy remains a challenge in contemporary practice. Even surgical revascularization of diffuse coronary artery disease is associated with poor one-year outcomes, as Shiono et al. demonstrated frequent (26%) occlusions of internal mammary artery grafts to functionally significant, diffuse LAD disease [21]. PCI optimization tools such as FFR and intravascular imaging help to reduce the occurrence of TVF compared to angiography-guided PCI, thus, their implementation, especially intravascular imaging, should be strongly encouraged. In addition, our data reveal that IVUS-optimized PCI to long diffusely diseased vessels reduces the frequency of residual myocardial ischemia as compared to functional optimization alone. Moreover, IVUS guidance is associated with a satisfactory one-year TVF rate even in the PCI of very long coronary stenoses. Therefore, future research comparing imaging guided and possibly hybrid PCI approach (DES and drug-coated balloons) to surgical revascularization is still needed in order to establish the best possible treatment modality for long diffuse coronary artery disease.

## 15. Limitations

It is important to interpret the findings of our trial with caution due to several limitations. Firstly, the study was conducted at a single center and lacked randomization, however, patients from both optimization strategies had very similar baseline clinical characteristics. Second, the sample size was relatively small, thus, it was not possible to ascertain statistically significant difference in the hard end-points. In order to achieve this a larger RCT trial is warranted. Third, employed IVUS optimization criteria for stent expansion might not be ideally suitable for long lesions, since it could overestimate stent expansion in the larger proximal part of the long lesion. Dividing the lesion length into two or three segments and evaluating expansion accordingly to those segments distal parts might be considered. However, the used IVUS console allowed performing only manual pull-back, therefore, it was not possible to estimate precise lesion length on IVUS and divide it into the segments accordingly. Fourth, invasive follow-up was not conducted for 18% of patients who declined further FFR measurements, although clinical follow-up was performed for all patients. Finally, the optimization rate and methods were not recorded for the FFR-optimized group.

## 16. Conclusions

In the treatment of long coronary artery lesions, the FFR PCI optimization strategy was associated with a higher incidence of poor functional PCI result and larger myocardial ischemia burden at follow-up compared to the IVUS optimization strategy. However, this discrepancy did not translate into a statistically significant difference in clinical outcomes. This study highlights the importance of using IVUS to optimize long lesions functional PCI outcomes.

## Figures and Tables

**Table 1:** Baseline clinical characteristics.

Clinical Characteristics	All Patients (*n* = 154)	FFR-Optimized Group (*n* = 74)	IVUS-Optimized Group (*n* = 80)	*p*
Age, years	66.3 ± 9.2	66.3 ± 9.6	66.2 ± 9.0	0.95
Male sex	111 (72.1)	54 (73.0)	57 (71.3)	0.81
Current smoker	35 (22.9)	15 (20.3)	20 (25.0)	0.51
Diabetes mellitus	31 (20.1)	16 (21.6)	15 (18.8)	0.66
Hypertension	141 (91.6)	67 (90.5)	74 (92.5)	0.66
Dyslipidemia	139 (90.8)	67 (90.5)	72 (91.1)	0.9
GFR < 60 mL/min/1.73 m^2^	28 (21.5)	12 (16.2)	16 (20.0)	0.59
Previous non-target-vessel-related myocardial infarction	75 (48.7)	29 (39.2)	46 (57.5)	0.02
Previous CABG	3 (1.9)	2 (2.7)	1 (1.3)	0.52
Number of diseased vessels				
Single-vessel disease	23 (14.9)	11 (14.9)	12 (15.0)	0.49
Two-vessel disease	74 (48.1)	39 (52.7)	35 (43.8)	
Triple-vessel disease	57 (37.0)	24 (32.4)	33 (41.3)	
Indications for PCI				
Chronic coronary syndrome	115 (74.7)	55 (74.3)	60 (75.0)	0.92
NSTE-ACS	39 (25.3)	19 (25.7)	20 (25.0)	
LV ejection fraction, %	55.0 [50.0–55.0]	50.0 [45.0–55.0]	55.0 [50.0–55.0]	0.02

Data are presented as mean ± standard deviation, median [Q1–Q3], and number (percentage). FFR—fractional flow reserve, IVUS—intravascular ultrasound, GFR—glomerular filtration rate, PCI—percutaneous coronary intervention, CABG—coronary artery bypass graft surgery, NSTE-ACS—acute coronary syndrome without ST segment elevation, LV—left ventricle.

**Table 2:** Angiographic and percutaneous coronary intervention related characteristics.

Procedural Characteristics	All Patients (*n* = 154)	FFR-Optimized Group (*n* = 74)	IVUS-Optimized Group (*n* = 80)	*p*
Target vessel				
LAD	127 (82.5)	61 (82.4)	66 (82.5)	0.83
LCx	15 (9.7)	8 (10.8)	7 (8.8)	
RCA	12 (7.8)	5 (6.8)	7 (8.8)	
PCI involving left main artery	15 (9.7)	2 (2.7)	13 (16.3)	0.01
Bifurcation two stent technique	10 (6.5)	3 (4.1)	7 (8.8)	0.24
Pre-dilatation	149 (96.8)	69 (93.2)	80 (100.0)	0.02
Largest pre-dilatation balloon diameter, mm	2.5 [2.5–2.75]	2.50 [2.2–2.8]	2.63 [2.5–2.9]	<0.001
Number of implanted stents	2 [1.0–2.0]	2 [1.0–2.0]	2 [1.0–2.0]	0.54
Total stent length, mm	56.0 [47.0–71.0]	49.0 [36.0–60.3]	62.5 [48.0–76.0]	<0.001
Total stent length ≥ 50 mm	89 (57.8)	36 (48.6)	53 (66.3)	0.03
Average stent diameter, mm	3.25 [3.0–3.5]	3.25 [3.0–3.5]	3.25 [3.0–3.5]	0.36
Average stent diameter > 3 mm	98 (63.6)	45 (60.8)	53 (66.3)	0.48
Maximal implantation pressure, atm	12.5 [12.0–15.0]	14.0 [13.9–16.0]	12.0 [11.5–12]	<0.001
Post-dilatation	143 (92.9)	63 (85.1)	80 (100.0)	<0.001
Maximal post-dilatation balloon diameter, mm	4.0 [3.5–4.0]	3.5 [3.5–3.5]	4.0 [3.8–4.5]	<0.001
Maximal post-dilatation pressure, atm	18.0 [16.0–20.0]	18.0 [16.0–20.0]	18.0 [16.0–20.0]	0.29
Contrast volume, ml	164 ± 51.8	162.3 ± 61.6	157.7 ± 41.4	0.84
Contrast induced nephropathy	0	0	0	

Data are presented as mean ± standard deviation, median [Q1–Q3], and number (percentage). FFR—fractional flow reserve, IVUS—intravascular ultrasound, LAD—left anterior descending artery, LCx—left circumflex artery, RCA—right coronary artery, PCI—percutaneous coronary intervention.

**Table 3:** Fractional flow reserve measurement immediately post-PCI and at 9–12 months follow-up.

FFR Characteristics	All Patients (*n* = 154)	FFR-Optimized Group (*n* = 74)	IVUS-Optimized Group (*n* = 80)	*p*
FFR_PRE PCI_	0.64 [0.54–07.0]	0.63 [0.52–0.69]	0.66 [0.59–0.71]	0.08
FFR_POST PCI_	0.88 [0.85–0.91]	0.88 [0.84–0.91]	0.88 [0.85–0.92]	0.6
ΔFFR_POST PCI_ and FFR_PRE PCI_	0.23 [0.17–0.33]	0.25 [0.19–0.36]	0.21 [0.16–0.31]	0.15
Stent gradient	0.06 [0.04–0.08]	0.06 [0.04–0.07]	0.07 [0.05–0.09]	0.06
Distal gradient	0.03 [0.02–0.06]	0.04 [0.02–0.07]	0.03 [0.01–0.05]	0.06
FFR follow-up performed	126 (81.8)	61 (82.4)	65 (81.3)	0.85
FFR_AT FOLLOW-UP_	0.87 [0.83–0.90]	0.87 [0.82–0.90]	0.87 [0.84–0.90]	0.47
ΔFFR_AT FOLLOW-UP_ and FFR_PRE PCI_	0.22 [0.17–0.33]	0.24 [0.15–0.36]	0.21 [0.17–0.28]	0.31
ΔFFR_AT FOLLOW-UP_ and FFR_POST PCI_	−0.01 [−0.04–0.01]	−0.01 [−0.04–0.01]	0 [−0.03–0.01]	0.46
Follow-up stent gradient	0.07 [0.04–0.09]	0.06 [0.04–0.08]	0.08 [0.05–0.10]	0.04
Follow-up distal gradient	0.03 [0.02–0.06]	0.04 [0.03–0.07]	0.03 [0.02–0.04]	0.02

Data are presented as median [Q1–Q3] and number (percentage). FFR—fractional flow reserve, IVUS—intravascular ultrasound, PCI—percutaneous coronary intervention.

**Table 4:** Intravascular ultrasound characteristics.

Characteristic	IVUS-Optimized Group (*n* = 80)
Number of IVUS runs	
2	50 (62.5)
3	27 (33.8)
4	3 (3.8)
Distal reference EEM diameter, mm	3.3 ± 0.5
Proximal reference EEM diameter, mm	4.6 ± 0.5
Minimal lumen diameter, mm	1.8 ± 0.2
Minimal lumen area, mm^2^	2.5 ± 0.6
Calcium arc ≥ 180°	39 (48.8)
Distal reference lumen area, mm^2^	5.9 ± 1.9
Distal reference EEM area, mm^2^	8.9 ± 3.3
Distal reference plaque burden, %	32.6 ± 9.2
Proximal reference lumen area, mm^2^	10.5 ± 2.8
Proximal reference EEM area, mm^2^	18.2 ± 4.1
Proximal reference plaque burden, %	42.0 ± 8.4
Minimal stent diameter, mm	2.5 ± 0.4
Minimal stent area, mm^2^	5.9 ± 1.9
Good stent expansion	73 (92.4)
Good stent apposition	79 (100.0)
No stent edge dissection	79 (100.0)
Plaque ≤ 50% near stent edges	56 (70.9)
Optimal IVUS result	54 (68.4)

Data are presented as mean ± standard deviation and number (percentage). IVUS—intravascular ultrasound; EEM—external elastic membrane.

**Table 5:** Comparison of medical treatment at discharge between patient groups with different PCI optimization strategy.

Medication	All Patients (*n* = 154)	FFR-Optimized Group (*n* = 74)	IVUS-Optimized Group (*n* = 80)	*p*
DAPT	143 (92.9)	72 (97.3)	71 (88.8)	0.01
OAC and antiplatelet	11 (7.1)	2 (2.7)	9 (11.2)	0.01
Statin	139 (90.8)	64 (87.7)	75 (93.8)	0.19
Beta-blocker	127 (83.0)	60 (82.2)	67 (83.8)	0.80
ACE-i/ARB	132 (86.3)	64 (87.7)	68 (85.0)	0.63

Data are presented as number (percentage). DAPT—double antiplatelet therapy; OAC—oral anticoagulant; ACE-I—angiotensin-converting enzyme inhibitor; ARB—angiotensin receptor blocker.

**Table 6:** Clinical outcomes of the study during 9–12 months follow-up.

Adverse Event	All Patients (*n* = 154)	FFR-Optimized Group (*n* = 74)	IVUS-Optimized Group (*n* = 80)	*p*
Functional TL restenosis	13 (10.9)	8 (15.1)	5 (7.6)	0.18
TV-related death	0	0	0	
TV-related MI	0	0	0	
TV revascularization	9 (5.8)	6 (8.1)	3 (3.8)	0.25
TV failure	9 (5.8)	6 (8.1)	3 (3.8)	0.25
Cardiac death	2 (1.3)	1 (1.4)	1 (1.3)	0.95
All-cause death	2 (1.3)	1 (1.4)	1 (1.3)	0.95

Data are presented as number (percentage). TL—target lesion, TV—target vessel, MI—myocardial infarction.

## Data Availability

All data can be shared by the authors upon reasonable request and in accordance with Lithuanian privacy regulations.
